# Allergenic Biomarkers in the Molecular Diagnosis of IgE-Mediated Wheat Allergy

**DOI:** 10.3390/ijms25158210

**Published:** 2024-07-27

**Authors:** Mariana Preda, Florin-Dan Popescu, Emilia Vassilopoulou, Sylwia Smolinska

**Affiliations:** 1 Department of Allergology “Nicolae Malaxa” Clinical Hospital, “Carol Davila” University of Medicine and Pharmacy, 022441 Bucharest, Romania; mariana.preda@umfcd.ro (M.P.); florindanpopescu@allergist.com (F.-D.P.); 2 Department of Nutritional Sciences and Dietetics, School of Health Science, International Hellenic University, 57400 Thessaloniki, Greece; vassilopoulouemilia@gmail.com; 3 Department of Clinical Sciences and Community Health, Università degli Studi di Milano, 20122 Milan, Italy; 4 Department of Clinical Immunology, Faculty of Medicine, Wroclaw Medical University, 51-616 Wroclaw, Poland

**Keywords:** IgE-mediated wheat allergy, biomarkers

## Abstract

IgE-mediated wheat allergy can take on various forms, including childhood food allergy to wheat, wheat-dependent exercise-induced anaphylaxis in young adults, baker’s respiratory allergy/asthma in workers exposed to wheat flour inhalation, and contact urticaria that is caused by hydrolyzed wheat proteins in some cosmetics, and that is sometimes associated with a food allergy. Singleplex and multiplex immunoassays detect specific IgE antibodies to wheat allergenic molecular biomarkers such as omega-5 gliadin Tri a 19, lipid transfer protein Tri a 14, and alpha-amylase inhibitors. The fluorescence enzyme immunoassay with capsulated cellulose polymer solid-phase coupled allergens is a commonly used singleplex assay. Multiplex methods include the ELISA-based macroarray immunoassay using nano-bead technology and a microarray immunoassay on polymer-coated slides. Another promising diagnostic tool is the basophil activation test performed with omega-5 gliadin and other wheat protein types. Detailed comprehension of the structural and immunological features of the numerous wheat allergens significant in clinical settings is imperative for advancing diagnostic biomarkers for IgE-mediated wheat allergies.

## 1. Introduction

Hexaploid wheat (*Triticum aestivum*; Poaceae family, Pooideae subfamily, Triticeae tribe), known as common wheat, bread wheat, or soft wheat, is one of the most extensively cultivated cereals globally. It holds significant importance as a cereal, providing approximately 20% of the nutrient calories consumed by the world’s population. Common wheat is a main ingredient in bread, while the much less cultivated tetraploid durum wheat (*Triticum turgidum* ssp. *durum*), also known as ‘hard wheat’, is preferentially used for pasta and semolina. In the last century, the cultivation of ancient wheats, such as hexaploid spelt (*Triticum aestivum* ssp. *spelta*), tetraploid emmer (*Triticum dicoccum*), and diploid einkorn (*Triticum monococcum*), has been at significantly lower levels compared to that of the more prevalent modern wheat varieties [1,2,3].

Wheat grains are a valuable source of high-carbohydrate energy, essential nutrients, and dietary fibers. They play a crucial role in the human diet. Wheat grains contain a complex blend of gluten proteins, which are particularly important in wheat flour as they significantly influence the quality of bread. *Triticum* spp. versatile grains serve as a crucial source of roughly half of the global food calories consumed, providing essential proteins like gluten, minerals such as copper, magnesium, zinc, phosphorus, and iron, as well as vitamins from the B group, and vitamin E. Wheat is abundant in thiamine (vitamin B1), riboflavin (vitamin B2), niacin (vitamin B3), and dietary fibers and carbohydrates, all of which contribute to its nutritional value. The gluten protein network present in the cereal endosperm has unique rheological properties, such as viscosity, extensibility and elasticity, which are conferred by the major storage proteins, gliadins (classified as prolamins) and glutenins (classified as glutelins). Contrary to consumer expectations, common wheat has the lowest gluten and gliadin content, whereas ancient wheat is often perceived to have a lower gluten content. High glutenin content and a relatively low gliadins/glutenins ratio are key protein parameters associated with baking quality. Additionally, non-gluten wheat proteins with structural and metabolic functions have secondary roles in wheat quality. Amylase/trypsin inhibitors are abundant non-gluten wheat seed proteins with a crucial role in inhibiting the activities of nutrient-degrading enzymes, such as alpha-amylase (involved in starch degradation) and trypsin (involved in protein degradation), and in providing natural defense mechanisms against pests and pathogens in wheat. The concentrations of these inhibitors vary significantly among different types of ancient and modern wheat, with no indication of lower concentrations in older varieties of bread wheat compared to common bread wheat [1,2,3,4,5].

The main wheat- and gluten-related disorders are celiac disease (CD), dermatitis herpetiformis (Dh), non-celiac wheat sensitivity (NCWS), and wheat allergy (WA).

CD is an autoimmune disease that affects around 1% of people. It is characterized by chronic immune-mediated enteropathy and is triggered by the ingestion of dietary gluten and related prolamines in genetically predisposed individuals (HLA-DQ2 and HLA-DQ8 genes) [6,7,8,9]. CD is mediated by gliadin-specific Th1 cells, which induce chronic intestinal inflammation and damage to villi. This is associated with the generation of autoantibodies against the following tissue proteins: anti-tissue transglutaminase IgA, anti-endomysial IgA, and anti-deamidated gliadin peptides IgA and IgG [10]. Dh is a specific skin manifestation of CD, typical in adulthood, affecting approximately 13% of patients. CD has also been associated with chronic urticaria [11,12].

NCWS (less accurately named non-celiac “gluten sensitivity”) is a condition more common than CD, with an estimated prevalence of up to 6%. It is characterized by both intestinal and extra-intestinal manifestations related to the consumption of gluten-containing foods in the absence of a diagnosis of CD or WA (autoimmunity and IgE-mediated hypersensitivity are not pathogenetic mechanisms). Amylase/trypsin inhibitors in wheat activate the toll-like receptor TLR4-MD2-CD14 complex on intestinal innate immune cells, triggering the secretion of proinflammatory chemokines and cytokines. This process promotes intestinal and extra-intestinal inflammation, contributing to the symptoms of NCWS [9,13,14].

WA manifests through immunologic hypersensitivity reactions to wheat proteins. Diseases in which wheat often serves as a common trigger, such as food protein-induced enterocolitis syndrome (FPIES) and food protein-induced enteropathy (FPE) in infancy and childhood represent non-IgE-mediated food allergy disorders, while eosinophilic esophagitis (EoE) in pediatric and adult patients is considered a mixed-IgE and non-IgE-mediated food allergy. The pathogenesis of non-IgE-mediated food allergy needs to be more adequately comprehended; however, circulating specific IgE antibodies are notably absent. Although cellular immunity mechanisms, mainly specific T cell responses, are considered important in driving inflammatory reactions, lymphocyte transformation tests (LTT) to wheat or other foods remain experimental and are not recommended [15,16].

In contrast, IgE-mediated WA involves specific IgE antibodies against wheat allergens, which play a central role in activating mast cells and basophils through cross-linking. This type of WA is defined by typical symptoms that usually develop within 2 hours of exposure to the allergen and are reproducible upon re-exposure, and evidence of IgE sensitization and/or effector cell response to the culprit allergen. In patients with a history of suspected IgE-mediated WA, skin prick tests and/or the measurement of serum specific IgE are recommended as first-line tests in the diagnostic workup. Serum concentrations of specific IgE to wheat extracts reflect the amount of circulating IgE antibodies directed to the food allergen. Specific IgE to individual allergen components indicated as clinically relevant can be more specific than specific IgE to whole allergen extracts. When used in isolation, IgG and IgG4 values do not diagnose IgE-mediated WA. IgG4 antibodies are related to immune tolerance, and their presence is relevant to the frequency of ingestion and food tolerance. More research is needed to assess allergen-specific IgG4/IgE and allergen/total IgE ratios for the outcome of oral food challenges; further data on the diagnostic performance of the mast cell/basophil activation tests for wheat are needed [15,16,17].

## 2. The Importance of Accurate Diagnosis in IgE-Mediated Wheat Allergies

Wheat is commonly implicated in IgE-mediated food allergies, along with cow’s milk, hen’s eggs, soya, sesame, peanuts, tree nuts, shellfish, fish, legumes, vegetables, and fruits [17]. There are several different species and subspecies of wheat and more than 25,000 cultivars, but there are no clinically significant differences in allergenicity [18].

WA prevalence is estimated to be 0.1–4%. Previously published, overall pooled estimates of WA were 3.6% for lifetime self-reported prevalence, 0.7% for skin prick testing positivity, 3.9% for specific IgE positivity, and only 0.1% for objectively verified food challenges positivity. Generally considered to be approximately 1%, WA depends on geographic regions and the patient’s age [9,19].

Children have a higher prevalence of WA than adults, especially if wheat is introduced into the diet for the first time after six months of age; most outgrow this allergy by the age of 16. Gluten proteins, alpha-amylase inhibitors, and other wheat allergens trigger an IgE-mediated food allergy to wheat in youngsters. Most children allergic to wheat appear to suffer from moderate-to-severe atopic dermatitis, and wheat ingestion may elicit typical IgE-mediated reactions such as urticaria, angioedema, bronchoconstriction, gastrointestinal manifestations, and anaphylaxis [20,21,22].

In adults, food allergy to ingested wheat is infrequent. Although wheat-dependent exercise-induced anaphylaxis (WDEIA) is a rare IgE-mediated food allergy, it represents the most usual variant, with clinical manifestations resulting from the combination of wheat intake and physical exercise. Other cofactors are alcohol and acetylsalicylic acid/non-steroidal anti-inflammatory drugs; therefore, it may be considered as wheat-dependent cofactor-augmented anaphylaxis or a wheat allergy dependent on augmentation factors. WDEIA is triggered mainly by omega-5 gliadin Tri a 19 (ω5-gliadin allergy), but other wheat allergens may be involved in IgE sensitization such as the non-specific lipid transfer protein Tri a 14 [23,24]. The ingestion of wheat allergens alone is insufficient to trigger WDEIA. However, WDEIA occurs when physical exercise is performed after eating, leading to alterations in tissue transglutaminase enzymes localized beneath the gut epithelium. This may result in peptide aggregation, amplifying IgE cross-linking and triggering allergic reactions. Moreover, interleukin-6, actively produced during exercise in contracting skeletal muscles and peritendinous tissue, enhances the expression of tissue transglutaminase, further contributing to the onset of WDEIA [25,26,27,28].

The prevalence of WDEIA in adolescents and adults has been documented as ranging from 0.017% to 0.80%, irrespective of ethnicity or geographical region [29]. In addition, fewer cases of IgE-mediated WA were shown to develop after the diagnosis of CD. The wheat elimination diet in CD patients may result in the loss of immune tolerance, which can lead to the development of IgE sensitization. This can result in a range of clinical manifestations, including urticaria, angioedema, and even anaphylaxis [30].

IgE-mediated WA is not only a significant food allergy but also a notable occupational WA in bakers, known as bakers’ asthma (BA). It stands as the most common occupational allergy in various countries, impacting a considerable percentage of bakers, with a percentage as high as 40% in the UK. This type of respiratory allergy is characterized by elevated levels of specific IgE antibodies to various proteins, particularly amylase/trypsin inhibitors, as well as to gluten proteins. Interestingly, individuals with BA can tolerate the ingestion of wheat bread, and only a minority of these experience concomitant allergies to Pooideae grass pollen [31,32].

There are four important phenotypes of IgE-mediated WA [24,31], as follows:

- IgE-mediated food allergy to wheat, typically observed in children but rarely in adults, presents with various manifestations ranging from urticaria and angioedema to severe anaphylaxis and involves omega-5 gliadin Tri a 19, high-molecular-weight (HMW) glutenin, low-molecular-weight (LMW) glutenin, and alpha-amylase inhibitor allergens;
- Wheat-dependent exercise-induced anaphylaxis (WDEIA) or, more accurately, wheat allergy dependent on augmentation factors (WALDA), commonly seen among young adults, is characterized by omega-5 gliadin Tri a 19 as the most specific biomarker. Other wheat allergens involved include non-specific lipid transfer protein Tri a 14;
- Baker’s respiratory allergy/asthma, prevalent in workers exposed to the inhalation of wheat flour, features thiol reductase homologue Tri a 27 and dimeric alpha-amylase inhibitor Tri a 28 as significant biomarkers. A combination with higher diagnostic accuracy involves three additional allergens, tetrameric alpha-amylase inhibitor Tri a 29, 1-cys-peroxiredoxin Tri a 32, and serine protease inhibitor-like protein Tri a 39;
- Contact urticaria to wheat proteins, which occurs in adolescents and adults, is associated with the use of some cosmetics and sometimes with food allergies. This condition involves hydrolyzed wheat proteins (HWP)/gluten.

A precise diagnosis of an IgE-mediated wheat food and respiratory allergy is tremendously important, as avoidance is a critical step in clinical management, and no commercial products for allergen-specific immunotherapy are currently available [24,33]. Due to their low specificity, the use of specific IgE antibodies against native whole wheat extracts as serum allergy markers for clinical diagnosis is challenging. Because of cross-reactivity with other allergens, especially grasses, in vitro measurements of serum levels of IgE antibodies against whole wheat extracts may yield unreliable results. It is also worth mentioning that some allergen molecules may be underrepresented in natural extracts due to their relative insolubility [24]. Specific IgE antibodies to wheat proteins are frequently detected in the serum of atopic children of all ages, even in the absence of a genuine food allergy. Studies suggest that approximately 65% of individuals with grass pollen allergy may exhibit false-positive serum IgE to wheat [24,34]. The presence of specific IgE to wheat without a suggestive history of clinical manifestations upon wheat exposure is not a diagnostic marker because many individuals can present in vitro IgE sensitization to wheat and tolerate it, especially those with Pooideae pollen sensitization [35,36]. Cross-reactivity commonly occurs among herbaceous plants in the Poaceae family, particularly between grasses in the Pooideae subfamily (Poeae tribe) found in temperate regions and wheat (Triticeae tribe). This cross-reactivity can result in false-positive immunoassay results, especially in patients with IgE sensitization to grass pollen [37,38,39]. Additionally, in clinical practice, there are other challenging aspects regarding the use of specific IgE antibodies to wheat as a serum allergy biomarker. One such concern is that the levels predicting 95% of WA cases can be elevated, sometimes reaching as high as 100 kUA/L [34,39,40,41]. Furthermore, these serum levels of specific IgE antibodies to wheat may remain elevated even after pediatric patients have outgrown WA [21,42,43].

Although there are numerous well-characterized wheat allergenic molecules, such as gliadins, glutenins and alpha-amylase inhibitors, it remains challenging to select single major allergens or combinations of molecular allergens for component-resolved diagnosis in IgE-mediated WA [24].

## 3. Molecular Approach to IgE-Mediated Wheat Allergy and Allergenic Biomarkers

**Figure 1:**
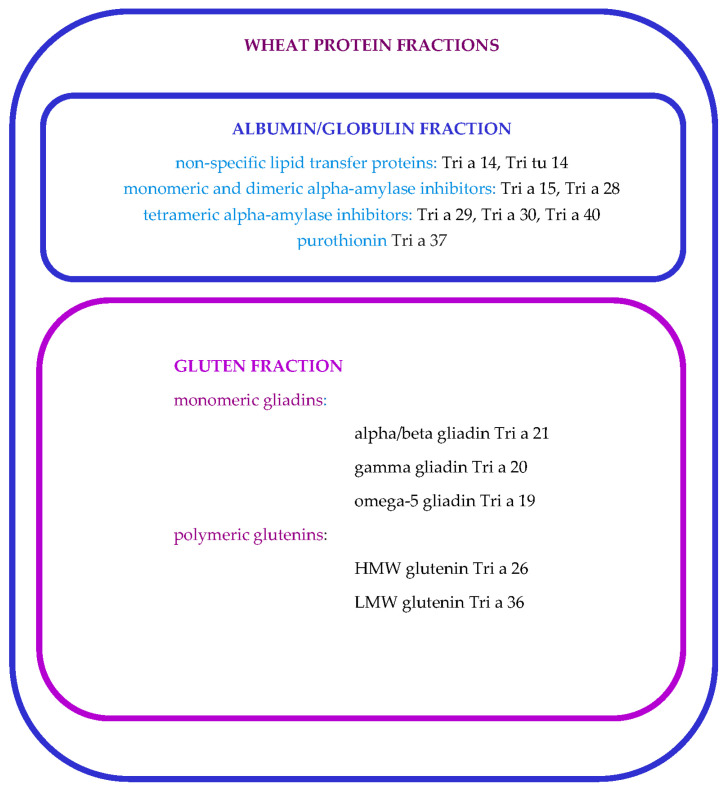
Important wheat allergenic molecules classification (adapted after [24,46,48]).

Wheat molecular components encompass some of the most complex proteins in nature, with proteomic analyses revealing up to 1300 molecules within its grains. Among these, specific proteins are recognized as allergens (Table 1) and can be categorized based on their solubility (Figure 1). The water/salt-soluble fraction, which includes albumins and globulins, contains allergenic proteins such as alpha-amylase inhibitors, avenin-like proteins, and lipid transfer proteins. Albumins are soluble in water and dilute salt solutions, while globulins dissolve in dilute salt solutions but not in water. Gluten proteins, constituting 70% to 80% of the total grain protein content, comprise the following two main groups: monomeric gliadins (including alpha/beta and gamma gliadins, omega-1,2, and omega-5 gliadins) and polymeric glutenins, consisting of high-molecular-weight (HMW) glutenins and low-molecular-weight (LMW) glutenins. Gliadins are soluble in aqueous alcohols (60% ethanol or 50% propanol) but insoluble in water and salt solutions. In contrast, polymeric glutenins, formed by HMW glutenin subunits linked by disulfide bonds to LMW glutenin subunits, are insoluble in these solvents but can be solubilized after a reduction of the bonds using 50% propanol-containing dithiothreitol [24,44,45].

**Table 1:** Characteristics of wheat molecular allergens mentioned in the World Health Organization/International Union of Immunological Societies database [24,46,47].

Allergen	Biochemical Designation (Other Characteristics)	Type of Exposure (Sensitization Comments)
Tri a 12	profilin (actin binding protein with low heat stability)	inhaled flour, food ingestion (2.5% BA pts)
Tri a 14	non-specific lipid transfer protein 1 (nsLTP-1 with high heat stability)	food ingestion, inhalation (involved in WDEIA, BA)
Tri tu 14	nsLTP-1	food ingestion (involved in WDEIA)
Tri a 15	monomeric alpha-amylase inhibitor 0.28 (high heat stability)	inhalation (10% BA pts)
Tri a 17	beta-amylase (maltohydrolase with high stability in acidic conditions)	food ingestion (41% WA pts)
Tri a 18	agglutinin isolectin 1 (carbohydrate binding lectin with high heat stability)	food ingestion (50–70% WA pts)
Tri a 19	omega-5 gliadin (seed storage protein with high heat stability)	food ingestion (80–90% WDEIA pts)
Tri a 20	gamma gliadin (low heat stability)	food ingestion
Tri a 21	alpha/beta gliadin	airway (50–70% WA pts)
Tri a 25	thioredoxin	food (50–70% WA pts)
Tri a 26	high-molecular-weight glutenin (high heat stability)	food (20% WDEIA; child WA)
Tri a 27	thiol reductase homologue (high heat stability)	airway (50–70% WA pts)
Tri a 28	dimeric alpha-amylase inhibitor 0.19 (high heat stability)	airway (37% WA pts)
Tri a 29	tetrameric alpha-amylase inhibitor CM1/CM2	airway
Tri a 30	tetrameric alpha-amylase inhibitor CM3	airway (high specificity BA)
Tri a 31	triosephosphate-isomerase	airway
Tri a 32	1-cys-peroxiredoxin	airway (60–80% WA pts)
Tri a 33	serpin (high heat stability)	airway ((involved in BA)
Tri a 34	glyceraldehyde-3-phosphate-dehydrogenase	airway (rarely involved in BA)
Tri a 35	dehydrin	airway (involved in BA)
Tri a 36	low-molecular-weight glutenin GluB3-2	food (60–80% WA pts)
Tri a 37	alpha purothionin	food (16% WA pts, severity)
Tri a 39	serine protease inhibitor-like protein	airway (involved in BA)
Tri a 40	tetrameric alpha-amylase inhibitor CM17	airway
Tri a 41	mitochondrial ubiquitin ligase activator of NFKB 1	airway
Tri a 42	tapetum determinant 1 (TPD1) fragment	airway
Tri a 43	transcriptional corepressor SCAI	airway
Tri a 44	endosperm transfer cell nsLTP-like protein	airway
Tri a 45	transcription elongation factor 1 (EIF1)	airway

The World Health Organization/International Union of Immunological Societies is commonly known by its acronym, WHO/IUIS. Tri a: molecular allergen from *Triticum aestivum* (wheat), Tri tu: molecular allergen from *Triticum turgidum durum* (durum wheat), WA: wheat allergy, pts: patients, CM: chloroform/methanol soluble protein.

The molecular allergy diagnostic approach to IgE-mediated WA uses allergen components in singleplex and multiplex immunoassays (Table 2). Given the heterogeneous nature of IgE responses in WA, it is important to consider multiple allergen components rather than relying on a single allergen molecule for accurate diagnosis. Currently, there are several commercially available wheat allergen molecules for in vitro diagnostic purposes: nGliadin, rTri a 19, rTri a 14 and nTri a aA_TI. The non-recombinant n-Gliadin is a natural mix of alpha-, beta-, gamma- and omega-gliadins (slow omega-gliadin and fast omega-gliadin), purified to 99%. rTri a 19 is the recombinant omega-5 gliadin, rTri a 14 is the recombinant non-specific lipid transfer protein, while nTri a aA_TI is the purified native alpha-amylase/trypsin inhibitor [24].

The fluorescence enzyme immunoassay (FEIA) with capsulated cellulose polymer solid-phase coupled allergens (ImmunoCAP^®^, Thermo Fisher Scientific Inc., Phadia AB, Uppsala, Sweden) is currently used as a solid-phase singleplex immunoassay to measure specific IgE antibodies to gliadin, Tri a 19 and Tri a 14. Molecular allergen components are covalently coupled in FEIA to a capsulated hydrophilic carrier polymer consisting of a cyanogen bromide-activated cellulosederivative with a large surface for protein binding. This method uses β-galactosidase-labeled anti-IgE monoclonal antibodies and 4-methylumbelliferyl-β-galactoside (MUG) as the fluorogenic substrate [49,51,52].

The ELISA-based macroarray immunoassay uses nano-bead technology as a molecular allergy explorer (ALEX^®^, MacroArray Diagnostics, Vienna, Austria) with allergen components spotted onto a nitrocellulose membrane in a cartridge chip using anti-human IgE labeled with alkaline phosphatase, and the microarray immunoassay on a preactivated amine-reactive polymer-coated glass slide as a solid phase uses fluorophore-labeled anti-human IgE monoclonal antibodies (Immuno CAP^®^ ISAC™, Thermo Fisher Scientific Inc., Phadia AB). Both are multiplex immunoassays able to measure specific IgE antibodies to Tri a 19, Tri a 14 and Tri a aA_TI. IgE sensitization to individual molecular allergens is associated with disease manifestations but with a significant overlap [23,49,50,51,52,53].

Quantitative enzyme-linked immunosorbent assays (ELISA) use immuno plates with hydrophilic surfaces, phosphate-buffered saline, and anti-human IgE monoclonal antibody conjugated with horseradish peroxidase (*HRP*) as a detection antibody, and tetramethylbenzidine as a substrate solution, and may be used to determine serum-specific IgE not only to omega-5 gliadin in WDEIA, but also to gamma-gliadin [54].

The Flow CAST^®^ (Cellular Allergy Stimulation Test) is the standardized and assessable basophil activation test (BAT) (Bühlmann Laboratories AG, Schönenbuch, Switzerland), which may be used for the quantitative assessment of in vitro basophil activation by wheat proteins, including omega-5 gliadin and HMW glutenin subunits, in whole blood samples by flow cytometry performed using the FACSCalibur^®^ system (Becton-Dickinson Immunocytometry System, Germany). CCR3 is used as an identification marker for basophils, while CD63 and CD203c are used as basophil activation markers, labelled with anti-CCR3-phycoerythrin, anti-CD63-fluorescein-isothiocyanate and anti-CD203c-phycoerythrin monoclonal antibodies, respectively. The BD CellQuest^®^ (Becton-Dickinson Immunocytometry System, Germany) is used to analyze data [48].

No single allergen molecule can be used solely for precise allergy diagnostics since the IgE responses in WA are heterogeneous and directed against multiple allergen components.

One of the best-characterized and commercially available wheat molecular allergenic biomarkers is the omega-5 gliadin Tri a 19, a seed storage protein mainly involved in WDEIA/WALDA but also important for early childhood IgE-mediated WA [24]. Gliadin, hordein, and secalin in wheat, barley, and rye, respectively, are alcohol-soluble storage proteins known as prolamins. According to electrophoretic mobility and allergenicity, fast omega-gliadin containing omega-5 gliadin is the major allergen among water/salt-insoluble proteins for WDEIA. Fast omega-gliadin presents cross-reactivity with gamma-gliadin and slow omega-gliadin. Furthermore, barley gamma-3 hordein, together with rye gamma-70 and gamma-35 secalins also cross-react with omega-5 gliadin. Therefore, Tri a 19 is considered a cross-reactive allergen with Tri a 20, Hor v 20, and Sec c 20 [55,56,57].

In patients with WDEIA, IgE sensitization to natural wheat extract is observed in only 20–30% of cases, whereas serum specific IgE antibodies to the allergenic molecule Tri a 19 are detected in 80–90% of patients. This disparity can be attributed to the fact that the gliadins responsible for WDEIA are insoluble in water and are not adequately present in aqueous wheat extracts. To circumvent this issue, the use of recombinantly produced Tri a 19 in immunoassays is needed for an accurate diagnosis [58].

This major sensitizing allergen molecule in WDEIA, along with the HMW glutenin Tri a 26 and the LMW glutenin Tri a 36, are relevant wheat allergens in food allergies but irrelevant for diagnosing BA. The specific IgE antibodies against Tri a 19 may be determined in serum by singleplex FEIA immunoassay and multiplex macroarray-based immunoassay; those against the isoform Tri a 19.0101 may be determined by multiplex microarray-based immunoassay, whereas those against Tria a 26 and Tria a 36 are not yet commercially available [24,59,60].

The primary IgE-binding epitope for the omega-5 gliadin has been identified through cDNA cloning. Specific IgE against epitope peptides of omega-5 gliadin may be helpful as research biomarkers for diagnosing WDEIA. Other major epitopes for HMW glutenins, and minor epitopes in alpha/beta-gliadins, gamma-gliadins, omega1,2-gliadins, and LMW glutenins, have been described [29,59,61,62,63].

Although IgE-mediated allergy to omega-5 gliadin Tri a 19 typically correlates with WDEIA, wherein physical exercise serves as a cofactor, some patients may present with idiopathic anaphylaxis, exercise-induced symptoms without an evident food connection, food-induced allergic manifestations without exercise, or recurrent acute urticaria [64]. Recently, it was suggested that adult patients experiencing intermittent acute urticaria should undergo screening for specific IgE sensitization to Tri a 19. This type of IgE sensitization has been observed in up to 22.5% of patients with more than one episode of acute urticaria over six months, not induced by physical factors, and not manifested daily and continuously for more than six weeks [65]. This is even more important because about 70% of WDEIA patients present with urticaria episodes before their first anaphylaxis [66].

Concentrations of serum IgE to rTri a 19 higher than a cut-off of 0.89 kU/L may confirm the WDEIA diagnosis with a 78–80% sensitivity and 96% specificity, while the specific IgE sensitivity for whole wheat extract and gluten is low, at 48% and 56%, respectively [21,67,68]. Other published data revealed that a cut-off value of 0.83 KU/L for specific IgE to omega-5 gliadin offers highly efficient diagnostic criteria for WDEIA, with a sensitivity of 89.3% and a specificity of 88.9% [69]. A suggested cutoff value of 0.3 of the logarithmically transformed specific IgE ratio of omega-5 gliadin to wheat has been proposed for enhanced diagnostic accuracy in adults with WDEIA. This calculated marker has demonstrated 100% sensitivity and specificity, surpassing the performance of individually specific IgE levels [70]. Moreover, BAT may differentiate between patients with WDEIA and control subjects using omega-5 gliadin and high-molecular-weight glutenin subunits as allergens [48,71].

It is important to note that Tri 19-negative patients with WDEIA can pose diagnostic challenges. In such cases, IgE sensitization to other wheat allergens is conceivable, including sensitization to the non-specific lipid transfer protein Tri a 14. Even though Tri a 14 is often involved in BA, few adult cases of Tri a 14-relevant IgE sensitization in WDEIA are reported, with cases reported even more rarely in children [72]. Tri a 14 from common wheat is a cross-reactive allergen with other non-specific lipid transfer proteins, such as durum wheat Tri tu 14, with which it shares about a 50% amino acid identity. This leads to a potential risk for cross-reactivity in WDEIA. Moreover, Tri a 14 and peach Pru p 3 have similar conformational regions involved in IgE binding, although their electrostatic features are different [2,5,73,74]. Sensitization to Tri tu 14 likely appears to be more important in wheat FA/WDEIA than in BA [2]. Apart from gluten and lipid transfer proteins, amylase/trypsin inhibitors may also play a role in WDEIA. Furthermore, BAT to non-gluten proteins carrying as yet unidentified allergenic epitopes may be useful as another tool in the precision diagnosis of WDEIA [75].

Omega-5 gliadin-specific IgE is also valuable for diagnosing immediate-type WA in children. A positive predictive value of 100% may be obtained using a cutoff of >5 kUA/L. Patients with higher specific IgE titers often exhibit more severe reactions. Omega-5 gliadin has been identified as an important allergen in children with wheat-induced anaphylaxis [70,76]. More recent data revealed that, in children, the level of specific IgE against Tri a 19 is a biomarker predictor of persistent WA in low-dose-tolerant patients (52 mg of wheat protein) stratified by an open-label oral food challenge [77]. IgE sensitization against rTri a 19 is detected in 20–30% of children with WA, and atopic dermatitis is detected in about 80% of pediatric patients with anaphylaxis after wheat ingestion. The presence of specific IgE antibodies to Tri a 14 is found in children with WA but these are not very sensitive. Even though it is currently considered that these do not cross-react with Poaceae pollen, there are not sufficient data to exclude this [21,34].

Regarding wheat IgE sensitization profiles to wheat allergens in children, in a very recently published Polish study, the molecules with the highest rates were Tri a A_TI (4.45%), followed by Tri a 14 (2.99%) and Tri a 19 (2.35%). The highest rates of positive specific IgE antibodies for all evaluated allergen molecules were observed in the <12-month-olds and the lowest in 13–18-year-olds [53]. A larger European study assessing exposome- and climate-dependent IgE sensitization profiles in children from different geographical regions used an EU-funded project multiplex immunoassay based on the MeDALL-allergen chip (mechanisms of the development of allergies) to assess the IgE-positive frequency to different wheat molecular allergens. The Norwegian cohort revealed specific IgE levels to Tri a 19.0101 similar to those in the Polish study, although they differed between age groups, with the highest specific IgE rates of 3.3% in 15–16-year-olds and lower rates in 7–12-year-olds (3%). In the Swedish cohort, the groups of 4-year-olds, 7–12-year-olds, and 15–16-year-olds had a steady Tri a 14-sensitization rate of 0.1%. Profilin Tri a 12 sensitization was higher in the Swedish 15–16-year-old group (2.5%) and Italian 7–12-year-old group (1.4%) than in the Norwegian cohort (0.4%). The frequencies of specific IgE against other individual wheat allergens (alpha-amylase/trypsin inhibitor Tri a aA_TI, glutathione-S-transferase Tri a GST, thioredoxin Tri a 25, 1-cys-peroxiredoxin Tri a 32, dehydrin Tri a 35, low-molecular-weight glutenin Tri a 36, alpha purothionin Tri a 37, serine protease inhibitor-like protein Tri a 39) varied across different European pediatric cohorts by between 0 to 1.2% [78]. Although the previously mentioned data are interesting from the molecular approach, for the in vitro component-resolved diagnosis of IgE-mediated WA in children, the only commercially available allergens are gliadin, omega-5 gliadin Tri a 19, non-specific lipid transfer protein Tri a 14, and alpha-amylase/trypsin inhibitor Tri a aA_TI.

Recent publications on systematic reviews and meta-analyses on the accuracy of in vitro diagnostic tests for IgE-mediated food allergy included studies of specific IgE to wheat and specific IgE to omega-5 gliadin, revealing pooled sensitivities of 72% and 79% and specificities of 79% and 78%, respectively. For specific IgE to wheat and to omega-5 gliadin, sensitivity is increased in subjects ≤ 16 years of age [33,79]. Some of these important studies were published in the last five years [39,80,81,82,83].

With the exception of BA, most food allergies correlate with unprocessed and processed wheat hypersensitivity. In the scientific literature, only a few cases of food allergy to specific unprocessed wheat were reported, mainly due to ingesting battered food, such as fish and chicken, which may contain raw wheat if only partially cooked. Such reactions seem to be due to IgE sensitization to the molecular gliadin allergen Tri a 21, correlated with positive tests for gliadin and gluten. Specific IgE against alpha/beta gliadin Tri a 21 was also reported to be an essential marker in WDEIA, even among patients negative to Tri a 19 [60,84,85].

Baker’s asthma (BA) and rhinitis are frequent work-related respiratory allergies; the causing agents are proteins mainly present in wheat flour, although enzymes used as additives in wheat and multigrain bread, including bacterial and fungal alpha-amylase, but also xylanase and cellulase, may also be involved. Wheat allergens are responsible for clinical manifestations in about 60–70% of bakers with the occupational allergy, and the molecular diagnostic approach to this type of IgE-mediated WA is progressively emerging [86,87,88].

Alpha-amylase inhibitors seem to be important allergens for BA. Dimeric alpha-amylase inhibitor Tri a 28, along with thiol reductase homologue Tri a 27, appear to be the most important biomarkers, but a combination with higher diagnostic accuracy involves the following three additional allergens: tetrameric alpha-amylase inhibitor Tri a 29, 1-cys-peroxiredoxin Tri a 32, and serine protease inhibitor-like protein Tri a 39. While the concurrent use of these five biomarkers shows promising diagnostic potential, it remains inferior to the detection of serum specific IgE antibodies against whole wheat flour extracts in BA. Adding Tri a 40 as a further wheat alpha-amylase inhibitor biomarker seems to have only a minimal influence on sensitivity and failed to improve diagnostic specificity. Moreover, two isoforms of Tri a 14 (Tri a 14.0101 and Tri a 14.0201) are considered minor allergens in BA. The nsLTP Tri a 14 and thioredoxin Tri a 25 share epitopes with Pooideae grass pollen allergens, but no such cross-reactivity was detected for alpha-amylase inhibitors Tri a 15 and Tri a 30, for alpha/beta-gliadin Tri a 21, or for serpin Tri a 31 [24,89,90,91,92]. A molecular allergy diagnostic approach may help to distinguish between IgE sensitization in BA, wheat FA, and wheat IgE seropositivity possibly with interference due to cross-reactivity to Pooideae grass pollen [2,31]. Two new allergenic proteins were recently reported as being involved in BA, a glucose/ribitol dehydrogenase and 16.9 kDa class I heat shock protein 1 [88].

Because many of the mentioned wheat individual allergens useful for occupational allergy diagnosis are not yet commercially available, use of the wheat extract is still the most important diagnostic option [24].

Immediate contact urticaria, with or without food hypersensitivity, attributed to the hydrolyzed wheat proteins (HWP) found in some cosmetic products, has been documented. In individuals sensitized to wheat protein hydrolysates, omega 5-gliadin appears to not be the major allergen. Instead, other gliadins or glutenins are suggested as being relevant [93].

HWPs are added to some cosmetics such as soaps, bath gels, body creams and hair-care products, and may induce IgE-mediated allergic contact urticaria [94,95,96]. Patients with an allergy to HWP tend to manifest as contact urticaria and HWP wheat-dependent exercise-induced anaphylaxis; the molecular allergens involved have not yet been clearly established [97]. Moreover, Asian cases of WDEIA associated with rhinoconjunctival IgE sensitization to HWP in facial soaps were reported, in some with the immunoassay detection of serum specific IgE to HWP (>25 kDa), salt-soluble wheat proteins (27–30 kDa) and salt-insoluble wheat proteins (30 kDa), but not to omega-5 gliadin Tri a 19 [93,98].

Using BAT in Japanese patients, HWP from soap enhanced CD203c expression in a concentration-dependent manner in patients with WDEIA sensitized by HWP (HWP-WDEIA). No significant enhancement of this basophil biomarker was observed with native or purified omega-5 gliadin, even if FEIA detects serum omega-5 gliadin-specific IgE. This indicates that specific IgE against HWP does not cross-react with omega-5 gliadin [99]. Published data in European patients indicate IgE sensitization to omega-1–2 gliadin and gamma-gliadin in this condition, with levels of IgE against omega-5 gliadin being relatively low only in some patients. This suggests that IgE sensitization to other gliadins (or glutenins) besides Tri a 19 might play a significant role in developing this phenotype. However, further data are required to elucidate this fully [100,101].

Finally, we must emphasize that cellular in vitro methods can be utilized to indirectly identify IgE-dependent sensitization to various wheat allergens. This can be done more accurately using BAT, as well as by a less reliable cellular allergen stimulation test that measures the release of sulfidoleukotrienes by blood basophils using ELISA [102,103,104]. BAT is not currently a routine diagnostic tool for WA as it is complex and expensive but may be useful for diagnosing food allergies in individual cases with unusually low total IgE or when specific IgE cannot be detected commercially against some molecular allergens such as HMW glutenin subunits [105,106]. In BAT, following cell stimulation and activation, basophils upregulate the expression of the activation surface markers that can be measured by flow cytometry such as CD63 (not expressed on resting basophils and related to anaphylactic degranulation) and CD203c (constitutively expressed on resting basophils and related to piecemeal degranulation). However, basophils downregulate membrane CD123 with activation in a subset of patients; therefore, while performing BAT, the use of gating strategies that depend solely on CD123 may lead to loss-to-analysis of basophils, particularly those that highly express CD63 and CD203c, resulting in a false-negative outcome in the test. The introduction of CD203c in the CD123/HLA-DR phenotyping protocol leads to a reduction in the loss of basophils in the gate and ameliorates the ability to catch basophils expressing CD63, thus preventing possible biases in the diagnostic value of BAT [104,107,108]. Other in vitro cellular tests based on basophil activation include active (aBHRA) and passive basophil histamine-release assays (pBHRA). In aBHRA, the amount of histamine released from activation is directly detected, while in pBHRA, the patient’s serum is used to sensitize basophils from a healthy donor from which the autologous IgE has been removed; histamine release is then quantified upon activation by allergen. Recently, BAT and aBHRA were assessed as valuable tools for identifying molecular sensitization profiles in WA, especially in WALDA [105].

It is imperative to underscore the substantial health impact of IgE-mediated WA at the global scale; therefore, the molecular perspective on allergenic components plays a pivotal role in unravelling IgE sensitization in WA, primarily in focusing on identifying and characterizing diagnostic biomarkers.

## 4. Conclusions

The accurate diagnosis of IgE-mediated wheat food and respiratory allergies is paramount, especially when considering the crucial role of avoidance in clinical management, particularly in the absence of commercially available products for allergen-specific immunotherapy. Various phenotypes are recognized within the spectrum of IgE-mediated WA, including a food allergy to wheat, wheat-dependent exercise-induced anaphylaxis or wheat allergy dependent on augmentation factors, baker’s asthma, and contact urticaria. Laboratory diagnostics primarily rely on singleplex and multiplex immunoassays to measure serum specific IgE antibodies directed against wheat allergenic biomarkers such as omega-5 gliadin (Tri a 19), lipid transfer protein (Tri a 14), and alpha-amylase inhibitors. Additionally, the basophil activation test targeting different wheat protein types emerges as a promising diagnostic tool. Given the heterogeneous nature of IgE responses in WA, it is imperative to consider multiple allergen components rather than relying on a single allergen molecule for accurate diagnosis. Challenges surrounding the purification and structural analysis of wheat proteins stem from their complex protein structure and solubility characteristics. A comprehensive understanding of wheat allergens’ significant structural and immunological features in clinical settings is essential. Such an understanding, coupled with the characterization of relevant IgE-binding epitopes across different types of WA, is pivotal for developing more effective diagnostic biomarkers tailored to IgE-mediated wheat allergies.

## Figures and Tables

**Table 2:** Wheat molecular allergenic biomarkers used in singleplex and multiplex IgE immunoassays [23,49,50].

Protein Family	Allergen	IgE Sensitization Biomarker
gliadins	Tri a 19	omega-5 gliadin or rTri a 19.0101 is a major allergen for wheat-dependent, exercise-induced anaphylaxis (WDEIA), an important allergen in early childhood wheat allergy (WA) with immediate onset symptoms and atopic eczema and baker’s asthma (BA)/allergy
non-specific lipid transfer proteins	Tri a 14	nsLTP-1 has obvious clinical relevance associated with baker’s asthma and food allergy
alpha-amylase/trypsin inhibitors	Tri a aA_TI	associated with both baker’s allergy and food allergy
